# *Slc20a2*-Deficient Mice Exhibit Multisystem Abnormalities and Impaired Spatial Learning Memory and Sensorimotor Gating but Normal Motor Coordination Abilities

**DOI:** 10.3389/fgene.2021.639935

**Published:** 2021-04-06

**Authors:** Yaqiong Ren, Yuqi Shen, Nuo Si, Shiqi Fan, Yi Zhang, Wanhai Xu, Lei Shi, Xue Zhang

**Affiliations:** ^1^McKusick-Zhang Center for Genetic Medicine, State Key Laboratory of Medical Molecular Biology, Institute of Basic Medical Sciences, Chinese Academy of Medical Science and Peking Union Medical College, Beijing, China; ^2^National Health Commission and Chinese Academy of Medical Sciences Key Laboratory of Molecular Probe and Targeted Theranostics, Harbin Medical University, Harbin, China

**Keywords:** primary familial brain calcification, PFBC, *Slc20a2*, vascular calcification, cognitive, sensorimotor gating, motor coordination

## Abstract

**Background:**

Primary familial brain calcification (PFBC, OMIM#213600), also known as Fahr’s disease, is a rare autosomal dominant or recessive neurodegenerative disorder characterized by bilateral and symmetrical microvascular calcifications affecting multiple brain regions, particularly the basal ganglia (globus pallidus, caudate nucleus, and putamen) and thalamus. The most common clinical manifestations include cognitive impairment, neuropsychiatric signs, and movement disorders. Loss-of-function mutations in *SLC20A2* are the major genetic causes of PFBC.

**Objective:**

This study aimed to investigate whether *Slc20a2* knockout mice could recapitulate the dynamic processes and patterns of brain calcification and neurological symptoms in patients with PFBC. We comprehensively evaluated brain calcifications and PFBC-related behavioral abnormalities in *Slc20a2*-deficient mice.

**Methods:**

Brain calcifications were analyzed using classic calcium-phosphate staining methods. The Morris water maze, Y-maze, and fear conditioning paradigms were used to evaluate long-term spatial learning memory, working memory, and episodic memory, respectively. Sensorimotor gating was mainly assessed using the prepulse inhibition of the startle reflex program. Spontaneous locomotor activity and motor coordination abilities were evaluated using the spontaneous activity chamber, cylinder test, accelerating rotor-rod, and narrowing balance beam tests.

**Results:**

*Slc20a2* homozygous knockout (*Slc20a2*-HO) mice showed congenital and global developmental delay, lean body mass, skeletal malformation, and a high proportion of unilateral or bilateral eye defects. Brain calcifications were detected in the hypothalamus, ventral thalamus, and midbrain early at postnatal day 80 in *Slc20a2*-HO mice, but were seldom found in *Slc20a2* heterozygous knockout (*Slc20a2*-HE) mice, even at extremely old age. *Slc20a2*-HO mice exhibited spatial learning memory impairments and sensorimotor gating deficits while exhibiting normal working and episodic memories. The general locomotor activity, motor balance, and coordination abilities were not statistically different between *Slc20a2*-HO and wild-type mice after adjusting for body weight, which was a major confounding factor in our motor function evaluations.

**Conclusion:**

The human PFBC-related phenotypes were highly similar to those in *Slc20a2*-HO mice. Therefore, *Slc20a2*-HO mice might be suitable for the future evaluation of neuropharmacological intervention strategies targeting cognitive and neuropsychiatric impairments.

## Introduction

Primary familial brain calcification (PFBC, OMIM#213600) is a rare autosomal dominant or recessive neurodegenerative and neuropsychiatric disorder characterized by bilateral symmetric cerebrovascular calcifications affecting multiple brain regions, particularly the globus pallidus, caudate putamen, and thalamus in humans (Wang et al., 2012; Saleem et al., 2013). *SLC20A2* encodes a ubiquitously expressed type III sodium-dependent phosphate cotransporter-2 protein (PIT-2) and acts as a gatekeeper for regulating phosphate (Pi) homeostasis in the cerebrospinal fluid (CSF) (Wallingford et al., 2017). *SLC20A2* mutations that impair Pi uptake are the major genetic causes of PFBC (Wang et al., 2012; Hsu et al., 2013). However, loss-of-function mutations in the *XPR1* gene, which encodes the only Pi-exporting transporter in eukaryotic cells, can also affect Pi homeostasis and lead to idiopathic brain calcification (Legati et al., 2015). *PDGFB–PDGFRB* signaling is essential for recruiting pericytes (PCs) and smooth muscle cells (SMCs) during angiogenesis (Lindahl et al., 1997; Hellstrom et al., 1999; Andrae et al., 2008), maintaining the normal brain vascular endothelial cell (EC) and microvessel morphology and function (Hellstrom et al., 2001), as well as blood–brain barrier (BBB) and neurovascular unit (NVU) integrity (Armulik et al., 2010; Daneman et al., 2010). Mutations in any of these genes that disrupt *PDGFB–PDGFRB* signaling are another genetic cause of idiopathic brain calcification, although BBB and NVU impairments are inconclusive for PFBC pathophysiology (Keller et al., 2013; Nicolas et al., 2013b; Villasenor et al., 2017; Jensen et al., 2018). *MYORG* and *JAM2* are two causative genes for autosomal recessive PFBC (Yao et al., 2018; Cen et al., 2020; Schottlaender et al., 2020), expressed mainly in astrocytes (ACs) and ECs, which are the fundamental NVU components (Vanlandewijck et al., 2018).

Excess CSF-Pi has been validated in patients with *SLC20A2* mutations (Paucar et al., 2017; Hozumi et al., 2018) and *Slc20a2* homozygous knockout (*Slc20a2*-HO) mice (Jensen et al., 2016; Wallingford et al., 2017). The *Slc20a2*-encoded inward Pi transporter is abundantly expressed on the ventricular side of the choroid plexus (CP) epithelium and SMCs of arterioles (Wallingford et al., 2017). These expression patterns imply that its functional deficiency might preclude Pi reflux from cerebral ventricles into blood vessels, resulting in ventricular Pi accumulation and the perivascular microenvironment of the brain parenchyma (Wallingford et al., 2017). Curiously, BBB integrity and permeability appeared normal in patients with *SLC20A2* mutations or even improved in the calcification-prone brain regions of *Slc20a2*-HO mice (Paucar et al., 2017; Wallingford et al., 2017; Jensen et al., 2018; Nahar et al., 2019).

Clinical and neurological symptoms in patients with PFBC do not occur in parallel with brain vascular calcifications (Donzuso et al., 2019; Grangeon et al., 2019). Neurobehavioral abnormalities usually emerge from mid-30 to mid-60 in patients and exacerbate with age; however, brain calcifications can be detected as early as adolescence. Broad clinical symptoms mainly include parkinsonism, tremor, dystonia, gait disturbance, dysarthria or other speech problems, chronic headache or dizziness, memory impairments, and other psychotic symptoms (Nicolas et al., 2013a; Grangeon et al., 2019; Guo et al., 2019). Patients with *SLC20A2* mutations are more susceptible to movement disorders, cognitive impairments, and psychiatric symptoms, especially parkinsonism and memory problems, compared with patients with PFBC caused by other gene mutations (Nicolas et al., 2013a, 2015; Donzuso et al., 2019; Grangeon et al., 2019).

*Slc20a2*-deficient mice are one of the most commonly used animals for studying pathophysiological PFBC mechanisms. Using *Slc20a2*-HO mice, the researchers had demonstrated that *Slc20a2* deficiency leads to elevated Pi levels in CSFs and proposed the “two-hit mechanism” hypothesis for brain calcification (Jensen et al., 2016; Wallingford et al., 2017). The intracellular nodules or calcified spots in astrocytes and pericytes (Jensen et al., 2018), SMC or EC impairment (Wallingford et al., 2017; Jensen et al., 2018), and the reactive astrocytes and activated microglia (Nahar et al., 2019; Zarb et al., 2019a, b) related with brain calcifications were all revealed using *Slc20a2*-PFBC mice. However, the neurobehavioral abnormalities corresponding to humans have not been evaluated in these mice, and this undefined understanding precludes us from designing and performing preclinical interventions and obtaining explanations.

## Materials and Methods

### Experimental Mice

*Slc20a2*^*t*m1a(EUCOMM)Wtsi^ gene-trapping mice with a universal knockout cassette were obtained from the European Mouse Mutant Archive (Munich, Germany; Skarnes et al., 2011)^1^. All *Slc20a2*-modified mice were maintained in SPF facilities under a 12 h light–dark cycle and provided free access to normal diet and clean water. The experimental *Slc20a2* mice were generated by breeding female *Slc20a2* heterozygous knockout (*Slc20a2*-HE) mice with male *Slc20a2*-HE mice at 2:1 mating ratio, producing wild-type (WT), *Slc20a2*-HE, and *Slc20a2*-HO offspring. Two primer pairs were used for *Slc20a2* genotyping, whose sequences were as follows: wt-for: 5′-tgccaaatgcccagatagtt-3′, wt-rev: 5′-gctggttgtgctgctaggtg-3′, tm = 60, wt size: 434 bp; mt-for: 5′-accggaaggagcaattcaag-3′, mt-rev: 5′-tcgtggtatcgttatgcgcc-3′, tm = 60, mt size: 249 bp.

### Quantitative Real-Time PCR

To confirm the knockout efficiency in *Slc20a2*-HO and *Slc20a2*-HE mice, we isolated cerebral microvessels as previously described (Takeda et al., 2010) and extracted total RNA using TRIzol^^TM^ reagent (Invitrogen, # 15596018) according to the manufacturer’s instructions. cDNA was synthesized using the SuperScript^^TM^ IV First-Strand Synthesis System (Invitrogen, # 18091050). Quantitative real-time PCR reactions were performed using LightCycler^®^ 480 SYBR Green I Master (Roche, # 4707516001) on a LightCycler^®^ 480 instrument. Each sample in the experimental and control groups was run in quadruplicate with two biological replicates. The relative expression was normalized to that of mouse *Gapdh* gene as a reference, and all samples were normalized to the WT group. The qPCR primers were as follows: m*Slc20a2*-for: 5′-ttcgtgtggctattcgtgtg-3′, m*Slc20a2*-rev: 5′-actttcctgaggctttcatcg-3′, tm = 60; m*Gapdh*-for: 5′-ggtgtgaacggatttggc-3′, m*Gapdh*-rev: 5′-gctcctggaagatggtgatg-3′, tm = 60.

### Single Photon Emission Computed Tomography

Single photon emission computed tomography (SPECT) was used to evaluate the skeletal morphology of *Slc20a2*-modified mice (WT, *Slc20a2*-HE, *Slc20a2*-HO). The experimental mice were anesthetized with isoflurane, and the images were captured using a micro-SPECT/CT camera (Bioscan, Nano SPECT/CT) with four rotating parallel hole collimators. All images were obtained in 360° rotation with 60 projections.

### Histopathological Staining

All experimental mice were perfused with 30 ml 0.01 M phosphate buffered saline (room temperature, pH 7.4) and 180 ml 4% ice-cold paraformaldehyde (PFA, pH 7.4) for 20 min through the left ventricular heart, followed by 4–20 h postfixation in 4% PFA in the dark on a 4°C rocking shaker. Whole brains were processed for routine dehydration in an ascending series of ethanol concentration and embedded in paraffin. Brain paraffin sections were cut into 2 μm-thick sections, dewaxed in xylene, and rehydrated through a descending series of ethanol concentration. The following staining procedures were conducted. After staining, the sections were dehydrated in ethanol, cleared in xylene, and mounted. Hematoxylin and eosin (H&E) staining was performed using standard procedures. Briefly, sections were rehydrated with water, stained with hematoxylin for 5 min, followed by washing with tap water. Then, 1% acid ethanol was added for 30 s and then rinsed in tap water for 30 min. Next, eosin solution was added for 1.5 min followed by tap water washing. The Alizarin Red S (ARS) method was used to display the calcium ions in the mouse brain. Sections were placed in ARS solution for 2 min, followed by dehydration and clearing in turn in acetone, acetone–xylene, and xylene. von Kossa (VK) staining detected the phosphate ion (PO_4_^3–^). After dewaxing and rehydration, 5% silver nitrate was added and exposed to ultraviolet rays for 45 min, after which the brain sections were rinsed in distilled water. The sections were then treated with 5% sodium thiosulfate for 10 min to wash the unreacted silver ions, followed by washing with distilled water. Next, nuclear fast red solution (Sigma-Aldrich, # N4638) was added for 5 min, and the sections were rinsed in distilled water.

### Behavioral Experiments

A standardized behavioral paradigm battery was applied on 8 month-old *Slc20a2*-modified mice. All behavioral assessments were performed at the Institute of Laboratory Animal Sciences, Chinese Academy of Medical Sciences. Before the experiment, the mice were properly handled and allowed to acclimatize to a slightly dark environment for 30 min. All instruments were cleaned with 75% ethanol between and after each evaluation.

### Morris Water Maze Test

The Morris Water Maze (MWM) test was based on the different distal shapes and colors of visual cues to navigate the mice for locating the hidden escape platform in a round black pool (120 cm in diameter) filled with opaque water at 25°C. The swimming pool was divided into four quadrants, and a submerged escape platform was placed in the northwest quadrant. The MWM paradigm consisted of five 60 s trials for each mouse per day for five consecutive days. Each mouse was semirandomly placed in different starting quadrants in daily navigation trials and allowed to locate the hidden platform in 60 s. In each trial, the trajectory of the mouse searching for the escape platform was recorded.

### Y-Maze Test

The Y-maze test was conducted using an instrument placed in a dim room consisting of three identical elevated arms (30, 8, and 15 cm in length, width, and height, respectively) placed at 120° to each other. The mice were individually placed at the end of one arm and allowed to independently explore the Y-maze for 8 min. The decision was considered to be correct if the mouse visited the three arms consecutively and wrong if one entered any individual arm more than once in three alternations. The percent alternative ratio was calculated as percent alternations (%) = [No. of right decisions/(No. of total arm entries − 2)] × 100% (Abdulbasit et al., 2018).

### Fear Conditioning

Fear conditioning was performed using a standard modular test chamber. On the training day, the mice were exposed to pure tone (volume: 80 dB, frequency: 5,000 Hz) and electric shock (current: 0.5 mA, duration: 1.0 s) pairings in 10 min. Each pure tone–electric shock session lasted 30 s with an electric shock in the last 1 s of the pure tone. The first session occurred at the 181st second, and each session was separated by 90 s. For contextual fear memory, the mice were placed in the same chambers with no tone or electric shock for 5.5 min. For cued fear memory on the third day, the inner chamber was filled with a black plastic baffle smeared with a sweet drink and the mice were exposed to pure tone at the same time point of the first day for 10 min. The freezing time of mice was recorded and analyzed.

### Prepulse Inhibition of the Startle Reflex

Sensorimotor gating capacity was assessed using the prepulse inhibition (PPI) paradigm, which presented a series of discrete trials comprising a mixture of four trial types including pulse-alone (120 dB), prepulse-alone (72, 76, or 84 dB), prepulse–pulse (72–120, 76–120, or 84–120 dB), and no-stimulus trials. Detailed PPI parameter settings are described in Supplementary Table 1. The startle reactivity (*S*) was reflected by the reactivity scores in the percentage score formula obtained from the pulse-alone trials (mean intensity of pulse-alone trials, excluding the first and last blocks of five consecutive pulse-alone trials). PPi*S* represents reactivity in prepulse–pulse trials (Ioannidou et al., 2018). Percentage score: PPI% = [(*S* − PPi*S*)/*S*] × 100% = {[pulse-only units − (prepulse–pulse units)]/(pulse–only units)} × 100%.

### Spontaneous Activity Chamber

The mice were individually placed in an activity chamber (approximately 20 cm in diameter) with six uniformly arranged infrared detectors for 10 min, and their activities were videotaped and stored for later analysis. The movements captured in turn by all infrared probes were defined as one active rotation. The number of active rotations in the last 5 min was analyzed.

### Cylinder Test

Mice were gently placed individually in a transparent plastic cylinder 10 cm in diameter and 20 cm in height. A digital recorder was used to capture all the spontaneous activities within 5 min. A mirror was placed on the opposite side of the video camera to capture all the behaviors. After the experiment, specific indices for motor function were counted and analyzed by replaying the video captured.

### Rotor-Rod Test

To assess motor balance and coordination, the mice were trained for four consecutive days on an accelerating rotarod with different maximum speeds, accelerated rates, and total test times. The apparatus consists of a rotating rod (diameter, 4.5 cm) divided into six sections by barriers with individual holding chambers below the rod. In probe trials, the mice were placed on the rod, which accelerated from 5 to 40 cycles per minute over a maximum of 300 s (day 01: 5–15 rpm over 20 s, total duration: 60 s; day 02: 5–30 rpm over 20 s, total duration: 60 s; day 03: 5–40 rpm over 90 s, total duration: 120 s; day 04: 5–40 rpm over 300 s, total duration: 300 s). The latency to fall from the rod into the holding chamber and the instantaneous falling speed of the rotating rod were recorded automatically. Each mouse was given a 15 min break and re-placed on the rod for the next trial. The mice were tested for six trials each day, and the mean latency and falling speed for each mouse were analyzed.

### Balance Beam Test

The narrowing balance beam test was also used to assess motor balance and coordination. The mice were trained on a wide crossbeam (9 mm wide, 75 cm long, 50 cm high) for the first 2 days. On the third day, they were placed on a narrow balance beam (5 mm wide, 80 cm long, 50 cm high) for crossing. The beam apparatus was housed in a dim environment, illuminated with 80-W light at the starting side to drive the mice to the opposite cage, recording the time crossing the beam and the number of hindfoot slips.

### Statistical Analysis

Gene expression and mouse behavioral test results were presented as individual mean ± SD and mean ± SEM. VK quantification was calculated as “(the positive VK staining areas divided by whole brain areas except bilateral olfactory bulb) × 100%” using Image-Pro Plus 6.0 software. MWM (time in target quadrant and swimming distance) and PPI (percentage score) were analyzed using two-way repeated measures analysis of variance (ANOVA) followed by simple effects analysis when the two-factor interaction existed. The swimming speed in the MWM test and the number of active rotations in the activity chamber were analyzed using one-way ANOVA. Tukey’s *post hoc* test was performed after two-way and one-way ANOVA. For rotor-rod and balance beam tests, daily performance was successively compared between *Slc20a2*-HO and WT mice by unpaired *t*-test considering the different experiment parameters in each day. The influence of body weight in these two assessments was evaluated and eliminated using covariance and linear regression analyses. Y-maze, fear conditioning, and cylinder test results were also analyzed by unpaired *t*-test. A standard *t*-test was performed when the data satisfied an approximate normal distribution and homogeneity of variance; otherwise, Welch’s *t*-test was used for data that only satisfied approximate normality, and the Mann–Whitney *U*-test was used for data that deviated from normal distribution. The *t*-test was executed on GraphPad Prism 7.0, and the other analyses were performed using IBM SPSS Statistics software (version 24.0). Statistical significance was set at *p* < 0.05. ^∗^, ^∗∗^, ^∗∗∗^, ^****^, and n.s. represent *p* < 0.05, *p* < 0.01, *p* < 0.001, *p* < 0.0001, and not statistically significant, respectively.

## Results

### Developmental Delay and Multisystem Abnormalities in *Slc20a2*-HO Mice

**FIGURE 1:**
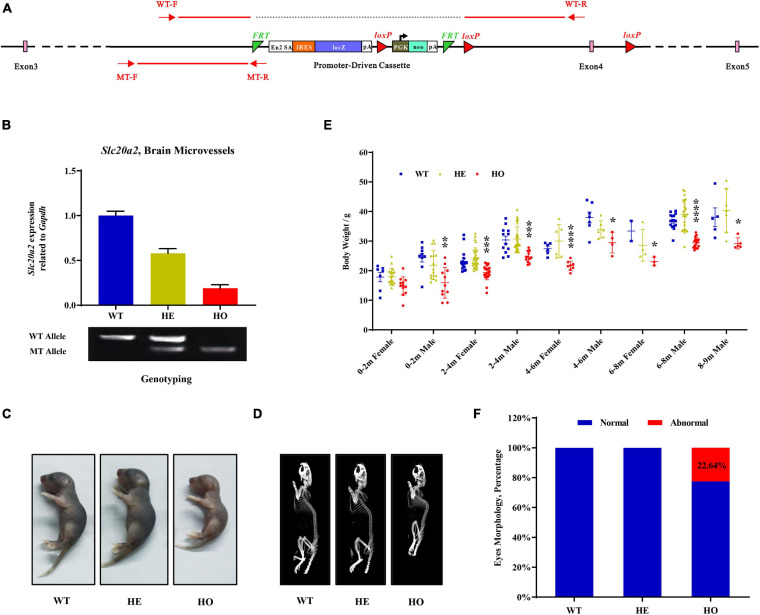
The overall appearances of *Slc20a2*-deficient mice. **(A)** *Slc20a2* gene-trapping cassette. Red arrows and lines indicate the genotyping primer location and PCR amplicon sequence in wild (434 bp) and mutant (249 bp) alleles. **(B)** *Slc20a2* mRNA expression in the brain microvessels of genotype-defined mice. **(C)** Overall appearances of 4-day-old mice. **(D)** Computed tomography (CT) imaging of 8 month-old mice skeleton. **(E)** The summary of body weight of mice with different ages, sex, and genotypes. Each symbol represents an individual. **(F)** The proportion of eye abnormalities in adult mice with different genotypes. *p < 0.05; **p < 0.01; ***p < 0.001; ****p < 0.0001.

The *Slc20a2* gene-trapping cassette for targeting and generating *Slc20a2*-HO and *Slc20a2*-HE mice is shown in Figure 1A. WT-F/WT-R and MT-F/MT-R primer pairs were used to amplify the WT (434 bp) and trapped allele (249 bp), respectively. To validate knockout or trapping efficiency, we analyzed *Slc20a2* expression in mouse cerebral microvessels using qPCR, which showed 19.02 and 57.99% relative *Slc20a2* expression in *Slc20a2*-HO and *Slc20a2*-HE mice, respectively, compared with that in WT mice (Figure 1B). Only *Slc20a2*-HO mice showed congenital and global developmental delay, lean body mass, and kyphosis (Figures 1C,D). The skeleton tended to be deformed in *Slc20a2*-HO mice, with a significant increase in physiological vertebral curvature (Figure 1D). We cross-sectionally surveyed and continuously recorded the weights of mice of different ages, sex, and genotypes and found that *Slc20a2*-HO mice had lower body weight than the mice with other two genotypes from birth to middle age (Figure 1E). Unilateral or bilateral eye defects, such as eyelid closure and microphthalmia, were observed in approximately 22.64% adult *Slc20a2*-HO mice (Figure 1F). Interestingly, the overall *Slc20a2*-HE mice appearance was similar to that of the WT mice despite haploinsufficient *Slc20a2* expression (Figure 1B) and high inorganic Pi concentration in the brain interstitial fluid.

### Patterns of Brain Vascular and Intracellular Calcifications in *Slc20a2-*HO Mice

**FIGURE 2:**
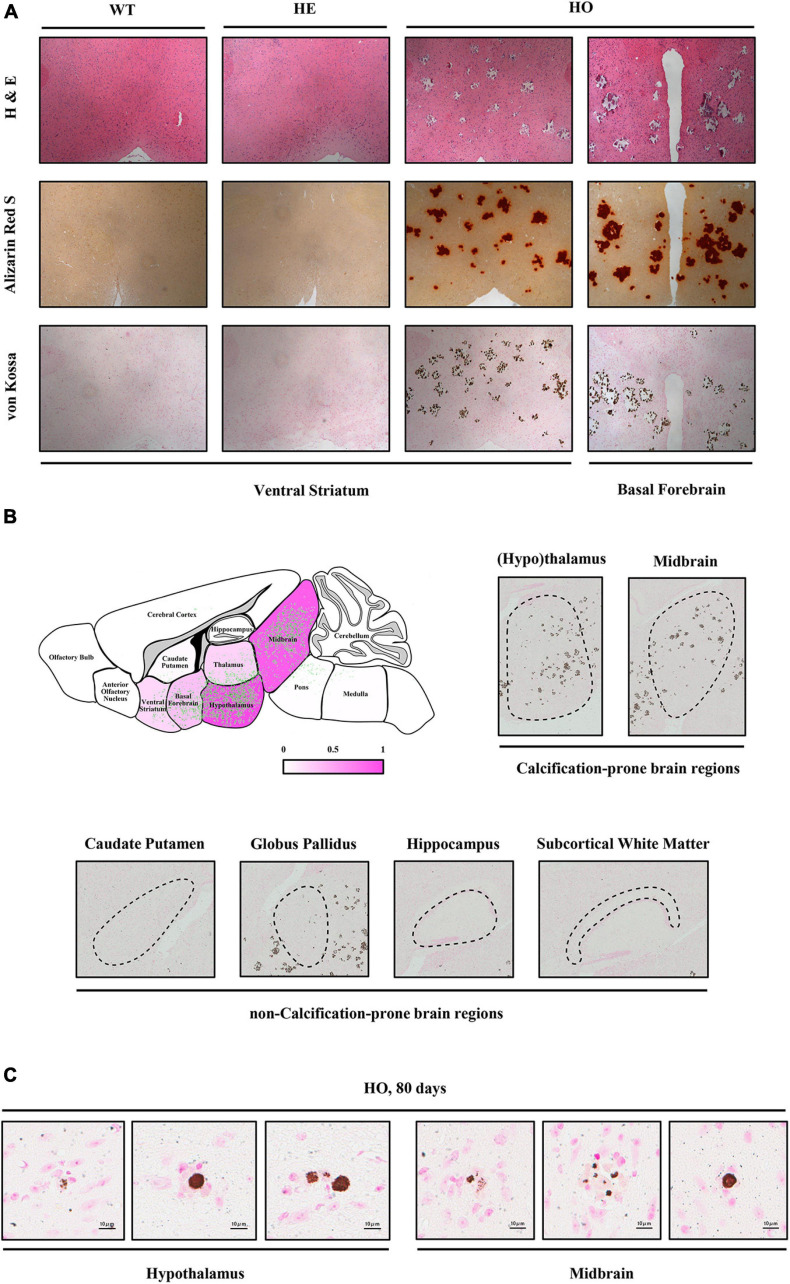
Histopathological characteristics of *Slc20a2*-deficient mice. **(A)** Calcium and phosphate staining on the coronal sections of different brain areas in genotype-defined mice. **(B)** Schematic diagram of calcification severity in different brain regions. Fuchsia and white represent calcification-prone and non-calcification-prone regions, respectively. Green dots indicate the distribution of brain calcifications from overlapping the actual calcified signals in 12 *Slc20a2*-HO brains. Representative pictures of VK staining on the calcification-prone and non-calcification-prone regions of the severely calcified brain of a 10 month-old mouse. Black dash circles indicate the target brain region. **(C)** Scattered intracellular calcified granules in calcification-prone brain regions of 80 day-old mice. Pink staining indicates cell nucleus and brown spots represent phosphate depositions.

To investigate the dynamic processes and patterns of calcification in *Slc20a2*-deficient mice, we stained the brain paraffin sections of different age–genotype *Slc20a2* mice groups using H&E, ARS, and VK. Only *Slc20a2*-HO mice showed extensive brain calcifications in the ventral striatum, basal forebrain, hypothalamus, thalamus, midbrain, and pons (Figures 2A,B), while other regions such as the caudate putamen, globus pallidus, hippocampus, subcortical white matter, cerebral cortex, and cerebellum were seldom involved (Figure 2B). In calcification-prone regions, we detected some tiny intracellular calcified granules or nodules and scattered and sporadic calcified spots as early as postnatal day 80 *Slc20a2*-HO mice (Figure 2C) and elderly HE mice, although this was a rare event.

### Impairment of Spatial Learning Memory and PPI in *Slc20a2*-HO Mice

Patients with *SLC20A2* mutation-induced PFBC usually exhibit several cognitive impairments and psychiatric symptoms after 40 years of age. To investigate whether *Slc20a2*-PFBC mice could recapitulate the neurological abnormalities presented in patients with PFBC, we evaluated four classic behavioral paradigms (MWM, Y-maze, contextual and cued fear conditioning, and PPI).

**FIGURE 3:**
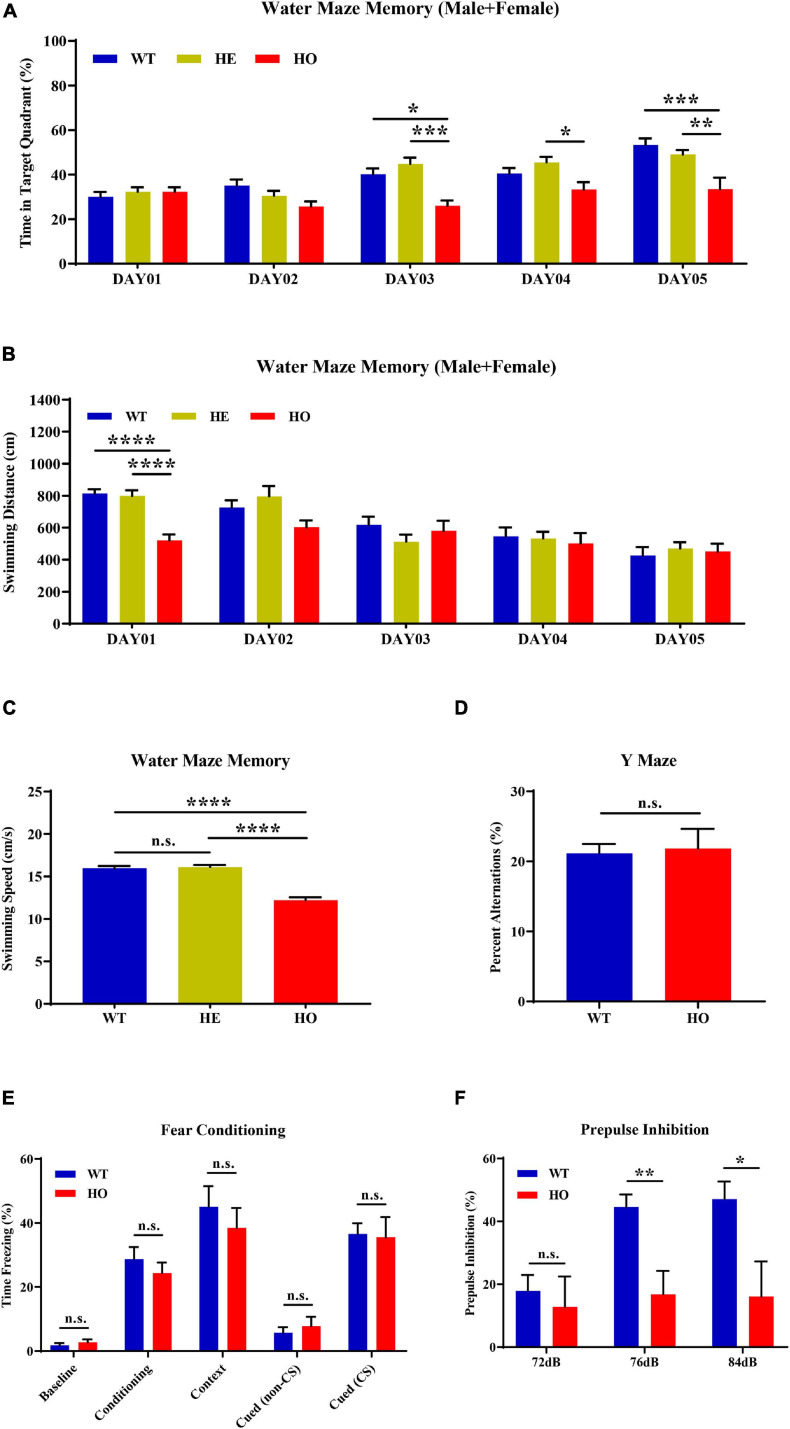
Spatial learning memory and prepulse inhibition (PPI) impairments in *Slc20a2*-deficient mice. **(A,B)** Spatial learning and memory ability revealed by the Morris water maze (MWM) test in the following dimensions: **(A)** spending time in the target quadrant (%) in each day trial. **(B)** Total swimming distance (cm) in each day trial. **(C)** Average mice swimming speed (cm/s) in all MWM experimental trials during 5 days. **(D)** The alternation score in spontaneous alternation Y-maze. **(E)** Fear memory revealed by freezing time (%) under different stimuli and environment. **(F)** The percentage score of PPI, reflecting the sensorimotor function of experimental mice. WT, wild *Slc20a2* genotype; HE and HO, heterozygous and homozygous *Slc20a2* gene trapping/knockout; *p < 0.05; **p < 0.01; ***p < 0.001; ****p < 0.0001; n.s., not statistically significant.

MWM is the most commonly used assessment tool for evaluating cognitive function related to spatial learning and memory in rodents. The procedure relies on distal shape and color cues to navigate mice from the start points around the perimeter of an open swimming arena to locate a submerged escape platform. From the percentage of swimming time in the platform quadrant summarized in all trials, *Slc20a2* genotypes, trial days, and their interactions showed a significant impact on the learning memory performance [two-way repeated measures ANOVA; for genotype, *F*_(__2_, _34__)_ = 9.118, *p* = 0.0007; for trial day, *F*_(__4_, _136__)_ = 17.540, *p* < 0.0001; for genotype–day interaction, *F*_(__8_, _136__)_ = 3.584, *p* = 0.0008; Figure 3A]. *Post hoc* analysis grouped by genotype showed that WT and *Slc20a2*-HE mice spent the same percentage of time in the target quadrant, but *Slc20a2*-HO mice were prone to navigate the non-specific arbitrary quadrants (*post hoc* Tukey’s test; WT vs. *Slc20a2*-HE: *p* = 0.948, *Slc20a2*-HE vs. *Slc20a2*-HO: *p* = 0.0008, WT vs. *Slc20a2*-HO: *p* = 0.002; Figure 3A). To overcome the effect of the cross term between genotype and trial day, we reanalyzed the simple effects of these independent variables. We found that (1) for the first and second trial days, “the percent of time in target quadrant” was not different among the three *Slc20a2* mice groups (for example, day 01: WT vs. *Slc20a2*-HE: *p* = 0.803, *Slc20a2*-HE vs. *Slc20a2*-HO: *p* = 1.000, WT vs. *Slc20a2*-HO: *p* = 0.898; Figure 3A). However, from the third to the fifth trial day, only *Slc20a2*-HO mice exhibited a relatively stable time percent in the target quadrant, reflecting difficulties to learn and recall the location of hidden underwater platform (for example, day 05: WT vs. *Slc20a2*-HE: *p* = 0.599, *Slc20a2*-HE vs. *Slc20a2*-HO: *p* = 0.007, WT vs. *Slc20a2*-HO: *p* = 0.0005; Figure 3A). (2) With the increase in training days, WT and *Slc20a2*-HE mice significantly increased the swimming time in the target quadrant (for example, day 05-WT vs. day 01-WT: *p* < 0.0001, day 05-*Slc20a2*-HE vs. day 01-*Slc20a2*-HE: *p* < 0.0001; Figure 3A), whereas the *Slc20a2*-HO mice had no improved performance in this navigation task after repeated training (*Slc20a2*-HO mice, multiple comparisons for each day, *p* > 0.05; Figure 3A). With respect to the total swimming distance in MWM, the training days, rather than genotypes, had an important role, as well as the interaction between the training days and genotypes in this dimension [two-way repeated measures ANOVA; for genotype, *F*_(__2_, _34__)_ = 1.661, *p* = 0.205; for trial day, *F*_(__4_, _136__)_ = 22.209, *p* < 0.0001; for genotype–day interaction, *F*_(__8_, _136__)_ = 3.279, *p* = 0.002; Figure 3B]. A simple effect analysis revealed that *Slc20a2*-HO mice swam less than the other two genotypes only in the day 01 trials (for example, day 01: WT vs. *Slc20a2*-HE: *p* = 0.980, *Slc20a2*-HE vs. *Slc20a2*-HO: *p* < 0.0001, WT vs. *Slc20a2*-HO: *p* < 0.0001; Figure 3B). The swimming speed of *Slc20a2*-HO mice was decreased compared with that of WT and *Slc20a2*-HE mice according to all experimental trials over 5 days (one-way ANOVA *post hoc* Tukey’s test; WT vs. *Slc20a2*-HE: *p* = 0.897, *Slc20a2*-HE vs. *Slc20a2*-HO: *p* < 0.0001, WT vs. *Slc20a2*-HO: *p* < 0.0001; Figure 3C).

The Y-maze assessment is widely used for evaluating working or short-term memory, rather than long-term spatial learning and memory, by monitoring the exploratory behaviors in rodents. In the Y-maze program, we did not detect a statistically significant difference in the spontaneous alternation percentage between *Slc20a2*-HO and WT mice (unpaired Student’s *t*-test; *t*_17_ = 0.234, *p* = 0.818; Figure 3D). Fear memory can be evaluated by the contextual and cued fear conditioning paradigm using freezing time as the fear response index in rodents. In the 3 day fear conditioning trials, the fear response did not change significantly between *Slc20a2*-HO and WT mice in the initial state (day 01, background noise without tone and electric shock; Mann–Whitney *U*-test, *U* = 27.000, *p* = 0.328; Figure 3E), after cued conditioning (day 01, a tone paired with an electric shock; unpaired Student’s *t*-test, *t*_16_ = 0.804, *p* = 0.433; Figure 3E), in a fear-based environment (day 02, previous environment, background noise without tone and electric shock; unpaired Student’s *t*-test, *t*_16_ = 0.691, *p* = 0.499; Figure 3E), and in a cued situation (day 03, new environment, without or with a tone stimulus alone; unpaired Student’s *t*-test, cued with non-CS: *t*_16_ = 0.659, *p* = 0.519, cued with CS: *t*_16_ = 0.163, *p* = 0.873, CS indicates tone stimulus; Figure 3E).

PPI is the suppression of a strong startle inducing reflex due to a weak preceded stimulus and is commonly used to evaluate the sensorimotor function for neuropsychiatric diseases, such as schizophrenia and mood disorders, in rodent models. *Slc20a2*-HO and WT mice had identical baseline startle responses (unpaired Student’s *t*-test; *t*_16_ = 1.510, *p* = 0.151, trials block1 + 2 + 3; Figure 3F). The intensities of startle stimulus, *Slc20a2* genotypes, and the intensity–genotype interaction significantly affected PPI [two-way repeated measures ANOVA; for genotype, *F*_(__1_, _14__)_ = 5.990, *p* = 0.028; for stimulus intensity, *F*_(__2_, _28__)_ = 8.762, *p* = 0.001; for genotype–intensity interaction, *F*_(__2_, _28__)_ = 5.227, *p* = 0.012; Figure 3F]. Under 76 and 84 dB startle stimuli, the *Slc20a2*-HO mice exhibited a weak suppression due to the preceding stimulus compared with that exhibited by WT mice (simple effect analysis; *Slc20a2*-HO vs. WT, 72 dB: *p* = 0.624; 76 dB: *p* = 0.003; 84 dB: *p* = 0.019; Figure 3F). In WT mice, the PPI percentage for 76 and 84 dB startle stimulus was significantly increased compared with that for 72 dB initial stimulus (simple effect analysis; 76 vs. 72 dB: *p* = 0.0006, 84 vs. 72 dB: *p* = 0.002, 84 vs. 76 dB: *p* = 0.954; Figure 3F). However, PPI was not significantly different in multiple comparisons of each stimulus in *Slc20a2*-HO mice (simple effect analysis: 76 vs. 72 dB: *p* = 0.889, 84 vs. 72 dB: *p* = 0.962, 84 vs. 76 dB: *p* = 0.999; Figure 3F).

MWM and PPI tests might be affected by sex or body weight differences. Body weight might not affect spatial learning memory (Golub and Germann, 2001; Iivonen et al., 2003; Vorhees and Williams, 2014) and sensorimotor gating processes (Dirks et al., 2003). In our experimental mice, body weight had a strong correlation with genotype and sex (Figure 1E), which was contrary to the requirement for independence of independent variables in multiple covariance analysis. We found a significant correlation between body weight and swimming distance [linear regression: day 01: *R*^2^ = 0.297, beta = 25.660, *F*_(__1_, _35__)_ = 14.760, *p* = 0.0005]. Subsequently, we performed mediation analysis to evaluate whether or to what extent the genotype was associated with swimming distance mediated by body weight in day 01 trials. The results showed that body weight partially mediated and contributed to 25.42% (mediation effect = *a* × *b*/*c*) of this association (Supplementary Figure 1A). Analysis of the swimming distance in day 01 trials stratified by sex indicated that both male and female *Slc20a2*-HO mice exhibited significantly reduced swimming distance during MWM evaluation (one-way ANOVA *post hoc* Tukey’s test for day 01; male: WT vs. *Slc20a2*-HE: *p* = 0.915, *Slc20a2*-HE vs. *Slc20a2*-HO: *p* = 0.0002, WT vs. *Slc20a2*-HO: *p* < 0.0001; female: WT vs. *Slc20a2*-HE: *p* = 0.983, *Slc20a2*-HE vs. *Slc20a2*-HO: *p* = 0.022, WT vs. *Slc20a2*-HO: *p* = 0.017; Supplementary Figure 1B). For “time in target quadrant” of the MWM test, only male *Slc20a2*-HO mice spent less time compared with *Slc20a2*-HE and WT mice in the hidden platform quadrant [two-way repeated measures ANOVA; male: for genotype, *F*_(__2_, _15__)_ = 6.846, *p* = 0.008; for trial day, *F*_(__4_, _60__)_ = 16.072, *p* < 0.0001; for genotype–day interaction, *F*_(__8_, _60__)_ = 4.220, *p* = 0.0005; female: for genotype, *F*_(__2_, _16__)_ = 2.269, *p* = 0.136; for trial day, *F*_(__4_, _64__)_ = 7.968, *p* < 0.0001; for genotype–day interaction, *F*_(__8_, _64__)_ = 2.223, *p* = 0.037; Supplementary Figure 1C]. Due to the lack of male *Slc20a2*-HO mice in the PPI test, we reanalyzed the female data and found that *Slc20a2*-HO mice had lower PPI response on 76 dB stimulus (simple effect analysis; 76 dB: WT vs. *Slc20a2*-HO, *p* = 0.020), whereas female *Slc20a2*-HO mice were less responsive to stimulation of any intensity (simple effect analysis; female *Slc20a2*-HO, 76 vs. 72 dB, *p* = 0.892; 84 vs. 72 dB, *p* = 0.967; 84 vs. 76 dB, *p* = 0.999; Supplementary Figure 1D).

### Normal Motor Coordination Abilities in *Slc20a2*-HO Mice

The most common neurological symptoms in patients with *SLC20A2* mutation are movement disorders, such as parkinsonism, tremor, dystonia, and gait disturbance. To comprehensively evaluate the general motor activity and motor coordination ability in the experimental PFBC mouse model, we employed a spontaneous activity chamber, cylinder test, accelerating rotor-rod test, and narrowing balance beam test to examine the abovementioned motor behavioral dimensions in *Slc20a2*-PFBC mice.

**FIGURE 4:**
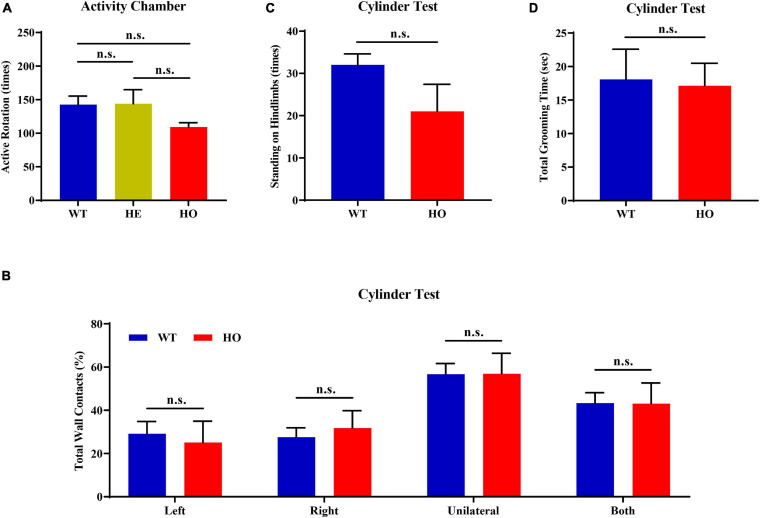
Normal motor coordination abilities revealed by the spontaneous activity chamber and cylinder test in *Slc20a2*-deficient mice. **(A)** Total active rotation number in the last 5 min in the activity chamber. **(B–D)** Cylinder test for locomotor asymmetry: **(B)** total number of wall contacts (proportion) using upper limbs, **(C)** standing times on hindlimbs, and **(D)** total grooming time. WT, wild *Slc20a2* genotype; HE and HO, heterozygous and homozygous *Slc20a2* gene trapping/knockout; n.s., not statistically significant.

First, we conducted an activity chamber test, a simplified assessment for determining gross locomotor activity in rodent models of CNS (central nervous system) disorders. The general locomotor activity had no statistically significant difference among the three groups of mice with distinct genotypes [one-way ANOVA; *F*_(__2_, _15__)_ = 1.037, *p* = 0.378; Figure 4A] and in multiple genotype comparisons (*post hoc* Tukey’s test; WT vs. *Slc20a2*-HE: *p* = 0.998, *Slc20a2*-HE vs. *Slc20a2*-HO: *p* = 0.403, WT vs. *Slc20a2*-HO: *p* = 0.429; Figure 4A). Only *Slc20a2*-HO mice showed a tendency to move less around the infrared detectors in the sound-attenuating and light-protecting chambers, reflecting a probable motor dysfunction. The cylinder paradigm is mainly designed to record and evaluate locomotor asymmetry, especially the spontaneous and asymmetric forelimb use in rodent models. *Slc20a2*-HO and WT mice showed comparable performance regardless of the independent use of their left (unpaired Student’s *t*-test; *t*_16_ = 0.386, *p* = 0.705) or right (unpaired Student’s *t*-test; *t*_16_ = 0.499, *p* = 0.625) forelimb and unilateral or bilateral (unpaired Student’s *t*-test; *t*_16_ = 0.016, *p* = 0.987) forelimbs when they reared against the cylinder wall (Figure 4B). The left-to-right ratios of independent forelimb use did not reach the statistical threshold between *Slc20a2*-HO and WT mice (Mann–Whitney *U*-test; *U* = 28.500, *p* = 0.375). The standing frequency of hindlimbs in contact with the wall and the cumulative duration of grooming behavior can partially reflect the anxious state and exploratory behavior in rodents. However, the number of hind paw stands (unpaired Student’s *t*-test; *t*_16_ = 1.831, *p* = 0.086; Figure 4C) and grooming duration (unpaired Student’s *t*-test; *t*_16_ = 0.151, *p* = 0.882; Figure 4D) were not significantly different between *Slc20a2*-HO and WT mice.

**FIGURE 5:**
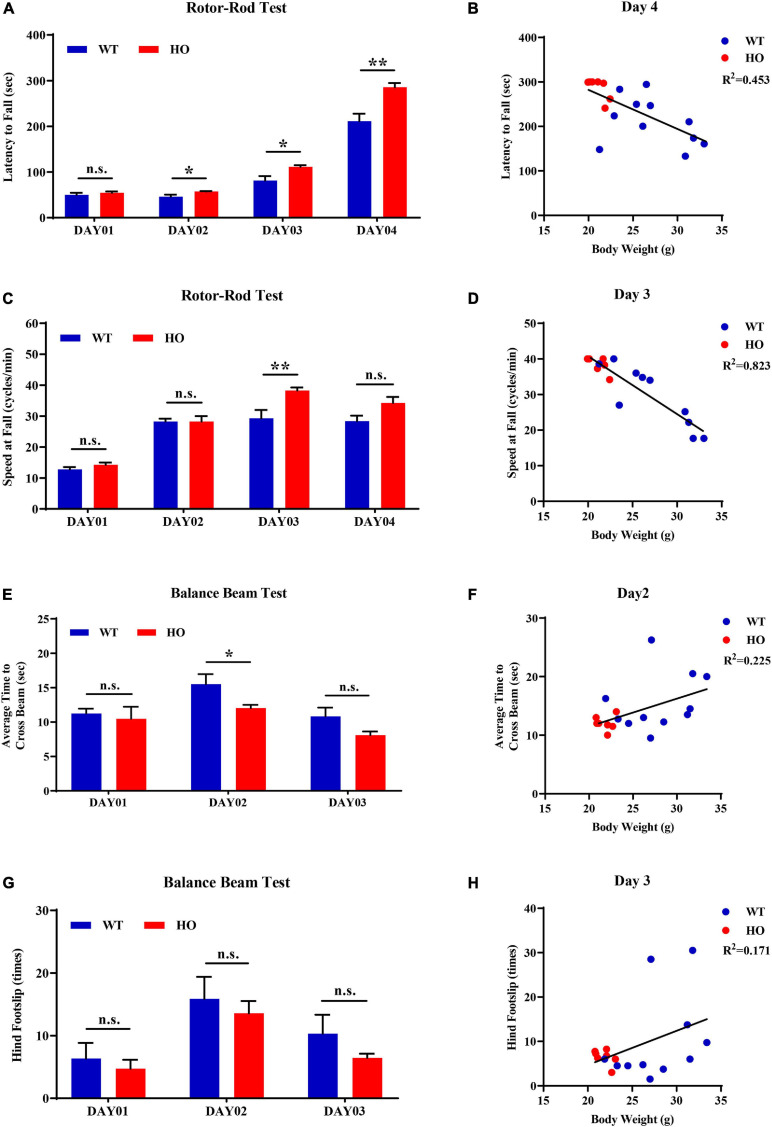
Normal motor coordination abilities revealed by accelerating rotor-rod and narrowing balance beam tests in *Slc20a2*-deficient mice. **(A–D)** Rotor-rod test for motor coordination: **(A)** time standing on the rotor-rod in each day trial. **(B)** Regression analysis for body weight and latency to fall in the day 04 trials of rotor-rod testing. **(C)** Falling speed of the rotor-rod in each day trial. **(D)** Regression analysis for body weight and falling speed in the day 03 trials of the rotor-rod test. **(E–H)** Balance beam test: **(E)** average time to cross the balance beam in each day trial. **(F)** Regression analysis for body weight and average time to cross the beam in the day 02 trials of balance beam test. **(G)** Hindfoot slips in each day trial. **(H)** Regression analysis for body weight and hindfoot slips in the day 03 trials of balance beam assessment. WT, wild *Slc20a2* genotype; HE and HO, heterozygous and homozygous *Slc20a2* gene trapping/knockout; *p < 0.05; **p < 0.01; n.s., not statistically significant.

The accelerating rotor-rod and narrowing balance beam tests are two classic motor function assessments for evaluating sensorimotor coordination, balance performance, and motor learning process. In the rotor-rod test, *Slc20a2*-HO mice performed significantly better than WT mice, as revealed by the increased time spent on the accelerating rotarod from day 02 to day 04 training and testing trials with constant rod diameter, start speed, and acceleration rate, as well as increased maximum speed and task difficulties (unpaired Student’s *t*-test or Welch’s *t*-test or Mann–Whitney *U*-test; *Slc20a2*-HO vs. WT, day 01: *t*_16_ = 0.737, *p* = 0.472; day 02: *t*_11_._66_ = 2.472, *p* = 0.030; day 03: *t*_12_._44_ = 2.852, *p* = 0.014; day 04: *U* = 6.000, *p* = 0.002; Figure 5A). Body weight was recognized as a common confounding factor affecting motor performance. This baseline difference in body weight also existed between *Slc20a2*-HO and WT mice (Welch’s *t*-test; *t*_11_._70_ = 4.925, *p* = 0.0004), and the regression analysis showed that the latency to fall was significantly negatively correlated with body weight in the day 04 trials (linear regression: *R*^2^ = 0.453, beta = -8.851, *F*_(__1_, _16__)_ = 13.250, *p* = 0.002; Figure 5B). To avoid this confounding factor, we adopted covariant analysis for staying time on the rotor-rod by controlling body weight, and the reanalyzed results showed that the performance in *Slc20a2*-HO was no longer better than that in WT mice for each day [one-way ANCOVA; *Slc20a2*-HO vs. WT, day 01: *F*_(__1_, _15__)_ = 0.991, *p* = 0.335; day 02: *F*_(__1_, _15__)_ = 0.938, *p* = 0.348; day 03: *F*_(__1_, _15__)_ = 2.286, *p* = 0.151; day 04: *F*_(__1_, _15__)_ = 1.818, *p* = 0.198]. For trials only on day 03, *Slc20a2*-HO mice had a significantly higher instantaneous falling velocity (Welch’s *t*-test; *t*_11_._05_ = 3.174, *p* = 0.009; Figure 5C), and the difference in falling speed disappeared by controlling body weight in the covariant analysis (one-way ANCOVA; *Slc20a2*-HO vs. WT, *F*_(__1_, _13__)_ = 0.508, *p* = 0.489). Linear regression analysis revealed a strong and negative correlation between the falling speed and body weight for trials on day 03 [linear regression: *R*^2^ = 0.823, beta = -1.625, *F*_(__1_, _14__)_ = 65.02, *p* < 0.0001; Figure 5D).

In the balance beam test, there was a slight statistical difference in the average time required for crossing the beam between two genotypes only in the day 02 trials (unpaired Student’s *t*-test or Welch’s *t*-test; *Slc20a2*-HO vs. WT, day 01: *t*_16_ = 0.477, *p* = 0.640; day 02: *t*_11_._94_ = 2.239, *p* = 0.045; day 03: *t*_12_._90_ = 1.956, *p* = 0.072; Figure 5E), whereas the number of hindfoot slips did not change significantly for each day (unpaired Student’s *t*-test or Mann–Whitney *U*-test; *Slc20a2*-HO vs. WT, day 01: *U* = 37.500, *p* = 0.930; day 02: *t*_16_ = 0.491, *p* = 0.630; day 03: *U* = 35.000, *p* = 0.791; Figure 5G). Body weight might not be an essential confounder in the balance beam test, as it was slightly positively correlated with the average time required for crossing the beam in the day 02 trials [linear regression: *R*^2^ = 0.225, beta = 0.472, *F*_(__1_, _16__)_ = 4.648, *p* = 0.047; Figure 5F] and showed no significant correlation with hindfoot slip number in the day 03 trials [linear regression: *R*^2^ = 0.171, beta = 0.783, *F*_(__1_, _16__)_ = 3.289, *p* = 0.089; Figure 5H].

For motor balance and coordination abilities, the basal ganglia with its subgroup nucleus and cerebellum are regarded as the most important brain regions, as well as the most frequently and severely calcified areas in patients with *SLC20A2* mutation, especially the caudate putamen and globus pallidus (Wang et al., 2012). Patients with *SLC20A2* mutation were more likely to be affected by movement disorders; however, they were barely seen in *Slc20a2*-HO mice, where the main functional regions for motor regulation were seldom calcified. Moreover, *Slc20a2*-HO mice had a better tendency to perform than WT mice in rotarod and balance beam tests before adjusting for body weight. The rotor-rod test might be the most susceptible for confounding body weight differences (Schellinck et al., 2010). The commonly used 3×Tg-AD mice with Alzheimer’s disease also perform well in the rotor-rod test, but perform poorly in other motor behavioral tasks, such as the balance beam test, suggesting the influence of body weight differences in middle-aged or older 3×Tg-AD mice (Stover et al., 2015; Garvock-de Montbrun et al., 2019). We also considered the potential impact of sex and reanalyzed four main dimensions in motor ability evaluations after sex stratification. Due to the insufficient number of male *Slc20a2*-HO mice in motor coordination assessments, only female mice were used for the stratified analysis. For “latency to fall” in rotor-rod test, female *Slc20a2*-HO mice showed superior performances only in the day 04 trials (unpaired Student’s *t*-test or Mann–Whitney *U*-test; female: day 01: *U* = 20.000, *p* = 0.620; day 02: *U* = 20.500, *p* = 0.620; day 03: *t*_12_ = 1.309, *p* = 0.215; day 04: *U* = 6.000, *p* = 0.017; Supplementary Figure 1E). Covariance analysis using body weight as a covariate also maintained a significant difference between female *Slc20a2*-HO and WT mice in holding time on the rotor-rod test [one-way ANCOVA; *Slc20a2-*HO vs. WT, *F*_(__1_, _11__)_ = 6.109, *p* = 0.031]. For falling speed in rotor-rod, as well as average time to cross the beam and hindfoot slips in the balance beam test, no significant differences were observed in each day trial in the female group (unpaired Student’s *t*-test or Welch’s *t*-test or Mann–Whitney *U*-test; speed at fall: day 01: *t*_5_ = 0.316, *p* = 0.765; day 02: *t*_6_ = 0.052, *p* = 0.960; day 03: *t*_10_ = 1.547, *p* = 0.153; day 04: *t*_9_ = 1.044, *p* = 0.324; average time to cross the beam: day 01: *t*_12_ = 0.037, *p* = 0.972; day 02: *U* = 15.500, *p* = 0.259; day 03: *t*_6_._84_ = 1.067, *p* = 0.322; hindfoot slips: day 01: *t*_12_ = 0.772, *p* = 0.455; day 02: *t*_12_ = 0.202, *p* = 0.844; day 03: *U* = 12.500, *p* = 0.128).

## Discussion

In this study, we comprehensively evaluated the multisystem abnormalities, brain calcification patterns, and neurobehavioral impairments in *Slc20a2*-deficient mice. *Slc20a2*-HO mice showed congenital and global developmental delay, lean body mass, skeletal malformation, and unilateral or bilateral eye defects. Brain calcifications were detected in the hypothalamus, thalamus, or midbrain early at postnatal day 80 in *Slc20a2*-HO mice, but seldom in *Slc20a2*-HE mice, even at an extremely old age. Brain calcifications might initiate with precalcified nodules and intracellular microcalcifications in *Slc20a2*-deficient mice. With age, extracellular calcifications accumulated and seemingly spread from initial locations such as the thalamus, hypothalamus, and midbrain to adjacent regions including the basal forebrain, ventral striatum, and pons in *Slc20a2*-HO mice. Only *Slc20a2*-HO mice exhibited spatial learning memory impairments and sensorimotor gating deficits, whereas their performance was absolutely normal in working and episodic memory tasks. Moreover, the general locomotor activity, motor balance, and coordination abilities showed no statistical differences between *Slc20a2*-HO and WT mice after adjusting for body weight, which was a major confounding factor in motor function evaluations.

### Gene Dosage Effect and Sensitivity in Mice and Patients With PFBC

*Slc20a2* encodes a type III sodium-dependent PIT-2 and acts as a gatekeeper in regulating Pi uptake and maintaining brain Pi homeostasis (Inden et al., 2016; Wallingford et al., 2017). *Slc20a2*-HO mice, with 19% relative *Slc20a2* gene expression, exhibited some unique overall appearances, such as developmental delay, skeleton malformation, cataract, or microphthalmia (Wallingford et al., 2016, 2017; Jensen et al., 2018; Beck-Cormier et al., 2019), which were not observed in patients with heterozygous *SLC20A2*, even barely in those harboring compound heterozygous or homozygous *SLC20A2* mutations (Wang et al., 2012; de Oliveira et al., 2013; Borges-Medeiros et al., 2019; Chen et al., 2019). Elevated Pi levels and severe calcification in the hypothalamus might induce neuroendocrine cell dysfunction, leading to hormonal deficiencies and growth delay in PFBC mice (Wu et al., 2018). Abnormal endochondral and intramembranous ossification and decreased mineral accumulation might be responsible for skeletal malformation (Beck-Cormier et al., 2019). Brain calcification in *Slc20a2*-HE mice has been extremely rarely reported, although *Slc20a2* expression was comparable to that observed in patients with *SLC20A2* mutation (Zhang et al., 2013). *Slc20a2*-HE mice, maintained in very few laboratories, with approximately 25% *Slc20a2* expression relative to that in WT mice, also simulate brain calcification phenotype in patients with PFBC (Wallingford et al., 2017). In patients with PFBC, *SLC20A2* gene dosage roughly correlates with the brain total calcification score (Wang et al., 2012; Chen et al., 2019) and partially reduces the Pi transport function of PIT-2 which might be insufficient to induce brain calcification (Nishii et al., 2019). Calcification degree and scope could be revealed by CT scan in the clinic, and these brain lesions had nearly complete penetrance for those harboring causative mutations, whereas its variable expressivity among patients with PFBC suggests the detrimental or protective effects of other genetic modifiers. Moreover, environmental factors could also affect the severity of brain calcification (de Oliveira et al., 2013; Donzuso et al., 2019; Grangeon et al., 2019; Borges-Medeiros and de Oliveira, 2020). The multisystem abnormalities and extensive brain calcifications found only in *Slc20a2*-HO mice might be explained by the discrepancy in gene dosage sensitivity between mice and humans.

### Pathological Process of Brain Calcification in Mice and Patients With PFBC

In *Slc20a2*-HO mice, the brain pathology might initiate as precalcified nodules and intracellular microcalcifications, which can be stably detected early at postnatal weeks 8–10 (Jensen et al., 2018); however, microcalcification emergence time in *Slc20a2*-HE mice depends on the actual *Slc20a2* expression dosage (Wallingford et al., 2017; Jensen et al., 2018). In the mouse brain, precalcified nodules contain polysaccharides and extracellular matrix, followed by phosphate and calcium component incorporation to form microcalcifications (Keller et al., 2013; Jensen et al., 2018). In the brains of patients with PFBC, microcalcifications can adhere to the capillary membrane and incorporate into or fuse with them, forming enlarged lamellar calcifications (Kobayashi et al., 1987; Miklossy et al., 2005). The most susceptible and severely calcified brain regions include the hypothalamus, thalamus, basal forebrain, ventral striatum, and midbrain in mice (Jensen et al., 2013, 2018; Wallingford et al., 2017), which are different from the basal ganglia (globus pallidus, caudate putamen, striatum), thalamus, subcortical white matter, cortex, cerebellum, and vermis commonly calcified in patients with PFBC (Wang et al., 2012; Nicolas et al., 2013a, 2015; Guo et al., 2019). Brain calcification spreading pathways and dynamic patterns revealed in *Slc20a2*-HO mice in this study were similar to those reported in a patient with pseudopseudohypoparathyroidism (Iwase et al., 2019), indicating a close relationship between the running courses of the cerebral deep penetrating arteries and brain calcifications.

### Neurobehavioral Abnormalities and Their Relations With Brain Calcifications

Clinical symptoms are complex among patients with PFBC with different gene mutations. Patients with *SLC20A2* mutation have a comparably high incidence of movement disorders, such as parkinsonism, accounting for approximately 70% of patients (Donzuso et al., 2019; Grangeon et al., 2019). However, *Slc20a2*-HO mice only exhibited long-term spatial learning memory impairments and sensorimotor gating deficits, as revealed by MWM and PPI tests, which corresponded with cognitive impairments and psychiatric signs observed in patients with *SLC20A2* mutation (Nicolas et al., 2013a, 2015; Donzuso et al., 2019; Guo et al., 2019). Motor coordination abilities were not assumed to be difficulties in *Slc20a2*-HO mice, suggesting that the neurobehavioral abnormalities in PFBC mice were partially parallel to the clinical observations in human patients.

The discrepancies in neurobehaviors might be related to the distinct calcification patterns in mice and humans with PFBC. Multiple brain regions can synergistically control and regulate various locomotor functions, such as motor learning, memory, performance, and coordination. The most important functional areas for their formation and regulation were the cerebellum (Nguyen-Vu et al., 2013; Lee et al., 2015; Darmohray et al., 2019; Nashef et al., 2019), motor cortex (Melzer et al., 2017; Nashef et al., 2019), dorsal thalamus (Parker et al., 2016), dorsal and lateral striatum, or basal ganglia, including the globus pallidus and caudate putamen (Durieux et al., 2009; Bateup et al., 2010; Kravitz et al., 2010; Borgkvist et al., 2015; Maurice et al., 2015), which are much less likely to calcify in *Slc20a2*-HO mice (Figure 2B). However, these regions, especially the globus pallidus, caudate putamen, and dorsal thalamus, were the first to be initiated and the most severely calcified brain regions in patients with PFBC (Nicolas et al., 2013a, 2015; Guo et al., 2019). Purkinje cell loss and neuroglial cell dysfunction in the cerebellum (Woo et al., 2018; Kana et al., 2019; Singh-Bains et al., 2019), as well as striatal inhibitory medium spiny neuron (MSN) (Durieux et al., 2009; Bateup et al., 2010; Guo et al., 2012), striatal cholinergic interneuron (Maurice et al., 2015; Melzer et al., 2017), and related striatal input or output neuronal circuit (Kravitz et al., 2010; Durieux et al., 2012; Borgkvist et al., 2015; Melzer et al., 2017) dysfunction could be the molecular and pathological motor impairment mechanisms. *Slc20a2*/PIT-2 is widely expressed in the cerebral and cerebellar neuronal cells, including NeuN^+^/Tuj1^+^ neurons in the cerebral and cerebellar cortex, Calbindin-D-28K^+^ striatal MSN neurons and cerebellar Purkinje cells, and TH^+^ substantia nigra or striatal neurons (Inden et al., 2013, 2016; Vanlandewijck et al., 2018), whose functional interaction with Map1b protein is essential for regulating cortical neuronal outgrowth (Ma et al., 2017). Neuron impairments, such as axonal loss, demyelination, and neuronal death, are uncommon in patients with *SLC20A2* mutation and *Slc20a2*-HO mice, unless in the advanced calcification stages and regions (Miklossy et al., 2005; Kimura et al., 2016; Jensen et al., 2018). The emergence of movement signs in patients with PFBC might be determined by the progressing calcifications in the cerebral cortex and cerebellum, or the neuronal impairments in the basal ganglia, in their late stages.

Long-term spatial learning memory impairments and sensorimotor gating defects in *Slc20a2*-HO mice were perfectly parallel with the cognitive impairments and neuropsychiatric symptoms in patients with PFBC. The hippocampus and dorsal striatum are the predominant structures involved in regulating spatial learning memory; however, these regions seldom have brain calcifications, suggesting that other areas are involved. Electrophysiological or pathophysiological abnormalities related to GABAergic hypothalamus–hippocampal CA3 projections (Wu et al., 2018; Zhou et al., 2019), ventral striatum–hippocampus neuronal circuits (Ferretti et al., 2010; Pennartz et al., 2011; Torromino et al., 2019), and cholinergic neurons in the basal forebrain (Martinez-Serrano et al., 1996), could also affect spatial learning processes and memory ability. These regions were highly calcified in the *Slc20a2*-HO mice (Figure 2B). Sensorimotor gating deficits are a hallmark of many schizophrenia-like symptoms in CNS disorders. Dopaminergic neurons in the hypothalamus (Barrios et al., 2020), cholinergic or non-cholinergic pedunculopontine tegmental nucleus neurons in the midbrain (Fulcher et al., 2020), and D2 receptor-expressing neurons in the nucleus accumbens (Shilling et al., 2006; Berger et al., 2011; Yamaguchi et al., 2015) could also be involved in sensorimotor regulation, and the functional impairments caused by brain calcification might be responsible for the defects in *Slc20a2*-HO mice.

The amygdala and prefrontal cortex are the two most essential regions for controlling fear (Ciocchi et al., 2010; Haubensak et al., 2010; Pape and Pare, 2010) and working memory (Rowe et al., 2000; Baeg et al., 2003; Curtis and D’Esposito, 2003; Stokes, 2015; Finn et al., 2019), respectively. Neuronal circuits between subdivisions of the amygdala and prefrontal cortex (Garcia et al., 1999; Adhikari et al., 2015; Jiang et al., 2016; Klavir et al., 2017) or dorsal/lateral thalamus (Penzo et al., 2015; Barsy et al., 2020) play a dominant role in fear memory acquisition and expression. Working memory is mainly related to the neuronal loops from the prefrontal cortex to the basal ganglia (Sawaguchi and Goldman-Rakic, 1991; McNab and Klingberg, 2008; Thorn et al., 2010). All the abovementioned regions were seldom calcified even at old age in *Slc20a2*-HO mice, in accordance with the normal performance in fear conditioning and Y-maze tests.

## Perspectives

In another PFBC mouse model, calcifications were frequently observed in the basal forebrain, thalamus, and midbrain of *Pdgfb*^ret/ret^ mice where the *Pdgfb* gene was hypomorphic (Keller et al., 2013). Zarb et al. demonstrated that *Pdgfb*^ret/ret^ mice exhibited sensorimotor gating deficits, hyperactivity, anxiety, and impaired working memory, recapitulating some neurological symptoms in patients with PFBC (Zarb et al., 2019b). No brain calcification was observed in *Jam2* gene knockout (*Jam2*-KO) mice; instead, the cerebral and cerebellar cortex, thalamus, and midbrain were prominently vacuolated (Schottlaender et al., 2020). *Jam2*-KO mice exhibited extensive reactive ACs, mild microglial activation, and reduction in neuronal density, as well as impaired motor coordination and gait disturbance in accordance with ataxia and parkinsonism in patients with PFBC (Schottlaender et al., 2020).

Two pathophysiological mechanisms, local calcium and phosphate homeostasis dysfunction and BBB–NVU impairment, might independently be involved in PFBC pathogenesis. BBB permeability and PC coverage of NVU showed no significant impairment in *Slc20a2*-HO mice and patients with *SLC20A2* mutation (Paucar et al., 2017; Wallingford et al., 2017; Jensen et al., 2018; Nahar et al., 2019). However, intravascular albumen components, such as IgG and fibrinogen, can leak into the brain parenchyma, such as the perivascular or paravascular spaces of calcified vessels (Miklossy et al., 2005; Jensen et al., 2018). The blood-borne leakage *via* intact BBB and PC might result from increased receptor-mediated transcytosis, although this has not been demonstrated in *Slc20a2*-deficient mice or patients with *SLC20A2* mutation (Villasenor et al., 2016; Yang et al., 2020). The impact of blood-borne substances on brain calcification remains unclear. An intriguing direction was related to brain-infiltrating and resident immune cells sensing a high Pi microenvironment before the emergence of brain calcification. In fact, CNS immune cells, such as microglia and astrocytes, are essential for brain calcification pathogenesis in mice and patients with PFBC (Keller et al., 2013; Nahar et al., 2019; Zarb et al., 2019a, b).

Considering that *SLC20A2* gene dosage might affect neurobehavioral impairments through brain calcification and dominant inheritance of patients with *SLC20A2*-PFBC, gene therapy for promoting WT allele expression might be a reasonable therapeutic option to manage this disease (Keasey et al., 2016; Matharu et al., 2019; Rabiee et al., 2020). Our study is the first to demonstrate that *Slc20a2*-HO mice could recapitulate some cognitive impairments and psychotic signs found in patients with PFBC, which might be beneficial for future design and preclinical interventions and explanations.

## Data Availability Statement

The original contributions presented in the study are included in the article/Supplementary Material, further inquiries can be directed to the corresponding author/s.

## Ethics Statement

The animal study was reviewed and approved by the Ethics Committee of Peking Union Medical College (PUMC) and the Institutional Review Board of the Chinese Academy of Medical Sciences (CAMS).

## Author Contributions

LS and XZ conceived and designed the study. LS, YR, and YS conducted most of the experiments and statistical analysis. LS and YR prepared the manuscript. LS, SF, and YZ checked and revised the grammar or syntax mistakes and logical errors. NS and YS provided technical guidance for the mouse behavioral assessments. SF, WX, and XZ offered clinical assistance and suggestions for interpreting the results of the pathological and behavioral experiments. All authors contributed to the article and approved the submitted version.

## Conflict of Interest

The authors declare that the research was conducted in the absence of any commercial or financial relationships that could be construed as a potential conflict of interest.

## Footnotes

1 www.infrafrontier.eu/search?keyword=EM:05549
