# Supplementary material for: Slc20a2-Deficient Mice Exhibit Multisystem Abnormalities and Impaired Spatial Learning Memory and Sensorimotor Gating but Normal Motor Coordination Abilities
Source: Front Genet. 2021 Apr 6;12:639935. doi: 10.3389/fgene.2021.639935 (PMC8056086; doi:10.3389/fgene.2021.639935)
Supplement: Supplementary file 1 [file Data_Sheet_1.docx]

Supplementary Material

# Supplementary Figures and Tables

## Supplementary Figure


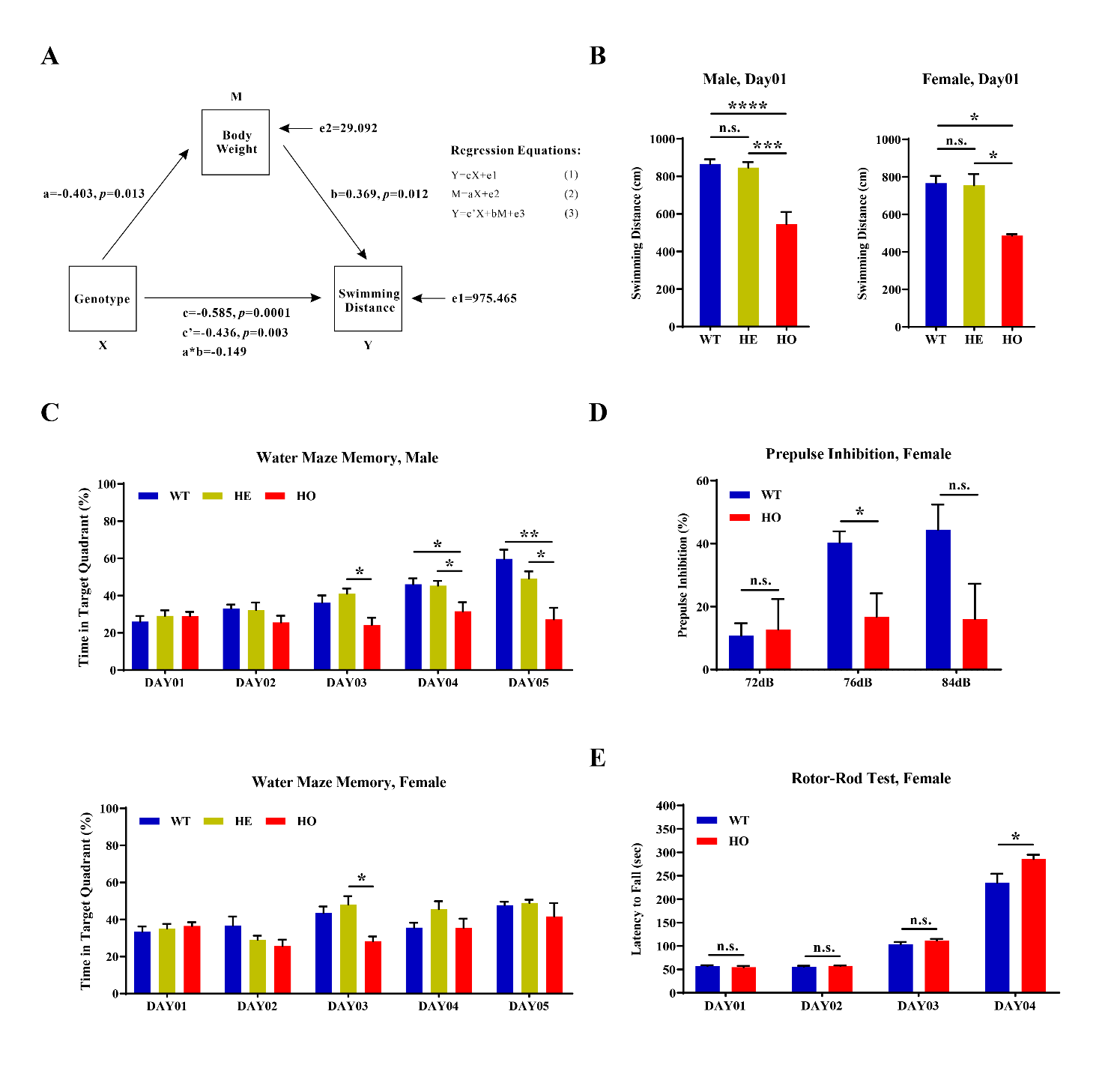


**Supplementary Figure S1 | Impact of body weight on spatial learning memory, and stratified analysis of Morris water maze (MWM), prepulse inhibition (PPI), and rotor-rod according to sex. (A)** Mediation analysis of the day-01 swimming distance in the MWM assessment. The coefficient **c** of equation (1): total effect of the independent variable (X) on the dependent variable (Y); the coefficient **a** of equation (2): the direct effect of X on intermediate variable (M); the coefficient **b** of equation (3): the direct effect of M on Y by controlling X; the coefficient **c′** of equation (3): the direct effect of X on Y by controlling M; the coefficient **a*b**: the mediation effect of M. **(B)** Stratified analysis of swimming distance according to sex in the MWM. **(C)** Stratified analysis of time in target quadrant according to sex in the MWM. **(D)** PPI test in WT and *Slc20a2*-HO female mice. **(E)** Rotor-rod test in WT and *Slc20a2*-HO female mice. WT, wild *Slc20a2* genotype; -HE and -HO, heterozygous and homozygous *Slc20a2* gene trapping/knockout; *, *p*<0.05; **, *p*<0.01; ***, *p*<0.001; ****, *p*<0.0001; n.s., not statistically significant.

## Supplementary Table

| **Supplementary Table 1 \| The parameter setting for PPI paradigm.** | | | | |
| --- | --- | --- | --- | --- |
| **Trial** | **Description** | **Repeat** | **Interval** | **Block** |
| Acclimation | Background，68 dB，5 mins |  |  |  |
| Pulse alone | 20 ms，120 dB | 5 Trials | 20s | Block I |
| Pulse alone | 20 ms，120 dB | 1 Trial × 10 | Randomly  10s ~ 30s | Block II |
| Prepulse | 20 ms，72 dB — 76 dB — 84 dB | 3 Trials × 10 |  |  |
| Prepulse + pulse | 20 ms prepulse — 100 ms Interval — 20 ms pulse | 3 Trials × 10 |  |  |
| NOSTIM | Background，68 dB | 1 Trial × 10 |  |  |
| Pulse alone | 20 ms，120 dB | 5 Trials | 20s | Block III |
